# Quantifying Recent Ecological Changes in Remote Lakes of North America and Greenland Using Sediment Diatom Assemblages

**DOI:** 10.1371/journal.pone.0010026

**Published:** 2010-04-02

**Authors:** William O. Hobbs, Richard J. Telford, H. John B. Birks, Jasmine E. Saros, Roderick R. O. Hazewinkel, Bianca B. Perren, Émilie Saulnier-Talbot, Alexander P. Wolfe

**Affiliations:** 1 Department of Earth and Atmospheric Sciences, University of Alberta, Edmonton, Alberta, Canada; 2 Department of Biology, University of Bergen, Bergen, Norway; 3 Bjerknes Centre for Climate Research, Bergen, Norway; 4 Environmental Change Research Centre, University College London, London, United Kingdom; 5 School of Biology and Ecology, University of Maine, Orono, Maine, United States of America; 6 Alberta Environment, Edmonton, Alberta, Canada; 7 Department of Geology, University of Toronto, Toronto, Ontario, Canada; 8 Department of Biology, McGill University, Montreal, Quebec, Canada; Northern Fisheries Centre, Australia

## Abstract

**Background:**

Although arctic lakes have responded sensitively to 20^th^-century climate change, it remains uncertain how these ecological transformations compare with alpine and montane-boreal counterparts over the same interval. Furthermore, it is unclear to what degree other forcings, including atmospheric deposition of anthropogenic reactive nitrogen (Nr), have participated in recent regime shifts. Diatom-based paleolimnological syntheses offer an effective tool for retrospective assessments of past and ongoing changes in remote lake ecosystems.

**Methodology/Principal Findings:**

We synthesized 52 dated sediment diatom records from lakes in western North America and west Greenland, spanning broad latitudinal and altitudinal gradients, and representing alpine (*n* = 15), arctic (*n* = 20), and forested boreal-montane (*n* = 17) ecosystems. Diatom compositional turnover (β-diversity) during the 20^th^ century was estimated using Detrended Canonical Correspondence Analysis (DCCA) for each site and compared, for cores with sufficiently robust chronologies, to both the 19^th^ century and the prior ∼250 years (Little Ice Age). For both arctic and alpine lakes, β-diversity during the 20^th^ century is significantly greater than the previous 350 years, and increases with both latitude and altitude. Because no correlation is apparent between 20^th^-century diatom β-diversity and any single physical or limnological parameter (including lake and catchment area, maximum depth, pH, conductivity, [NO_3_^−^], modeled Nr deposition, ambient summer and winter air temperatures, and modeled temperature trends 1948–2008), we used Principal Components Analysis (PCA) to summarize the amplitude of recent changes in relationship to lake pH, lake:catchment area ratio, modeled Nr deposition, and recent temperature trends.

**Conclusions/Significance:**

The ecological responses of remote lakes to post-industrial environmental changes are complex. However, two regions reveal concentrations of sites with elevated 20^th^-century diatom β-diversity: the Arctic where temperatures are increasing most rapidly, and mid-latitude alpine lakes impacted by high Nr deposition rates. We predict that remote lakes will continue to shift towards new ecological states in the Anthropocene, particularly in regions where these two forcings begin to intersect geographically.

## Introduction

There is mounting evidence that recent ecological and biogeochemical changes have occurred in remote lakes, defined here as those lacking any immediate, catchment-scale, anthropogenic influences. The implication is that these ecosystems can no longer be considered pristine, largely because of their high sensitivity to climate change [1], [2] which, in some regions, is compounded by significant inputs of reactive anthropogenic nitrogen (hereafter Nr; comprising all biologically-, photochemically- and radiatively- active nitrogenous compounds in the atmosphere and biosphere [3]) delivered by atmospheric deposition [4]–[6]. Part of the sensitivity of arctic and alpine lakes is attributable to limnological characteristics including dilute water chemistry, low primary production, and high flushing rates associated with nival hydrological regimes. The paucity of long-term climatic and environmental monitoring data in remote regions can be alleviated by the use of proxy data from high-resolution sedimentary records. Geochemical signatures and biological remains are continuously archived in the sediments accumulating at the bottoms of lakes. Diatoms (Bacillariophyceae) are unicellular aquatic photoautotrophs that respond rapidly to changes in water chemistry mediated by environmental change. Their siliceous cell walls are often preserved in lake sediments with sufficient fidelity to allow taxonomic identifications and ecological inferences, thus producing an archive of limnological history. In an evolving literature, sediment diatom records have been applied successfully to a range of global environmental issues including lake acidification [7], eutrophication [8], and climate change [2], [9]. Many of these data augment the evidence that the planet has entered the Anthropocene [10], [11], the era of human dominance over key biogeochemical cycles, with direct climatic and ecological repercussions.

**Figure 1:**
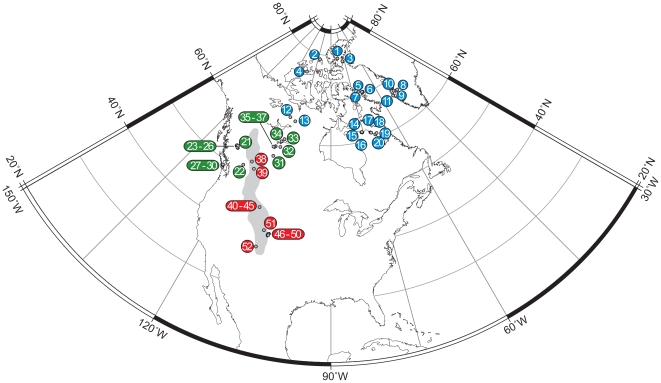
Location map of the study lakes in North America and Greenland. Numbers correspond to the descriptions in Table 1. Shaded area shows the extent of the Rocky Mountains.

In the present study we analyze the amount of diatom compositional turnover in sediments from 52 remote lakes in North America and west Greenland during three time intervals, each defined broadly by climate history: (1) the cold Little Ice Age (LIA), ∼1550–1800; (2) recovery following culmination of the LIA, 1800–1900; and (3) 20^th^ century warming. Geographically, the lakes span 38–79°N of latitude, and 12–3546 m asl of altitude, representing arctic (*n* = 20), alpine (*n* = 15), and forested boreal-montane (*n* = 17) ecosystems (Fig. 1, Table 1). The geographical gradients and temporal intervals captured by this sample array allow the sensitivity of remote lakes to be tested explicitly. The cornerstone of our analysis comprises estimates of diatom compositional turnover (or β-diversity) obtained by Detrended Canonical Correspondence Analysis (DCCA) constrained to time as the sole predictor variable [Materials and Methods, 12].

**Table 1:** Locations, physical characteristics, pH, conductivity, and β-diversity values from lakes in this study.

			Latitude	Longitude	Altitude	Lake area	Catchment	Z_max_	pH	Conductivity		β-diversity (SD units)		
Lake no.	Region	Lake name	(°N)	(°W)	(m asl)	(km^2^)	area (km^2^)	(m)		(µS cm^−1^)	20^th^ c.	19^th^ c.	1550–1800	Reference
**Arctic**														
1	Ellesmere Island	Sawtooth	79.33	81.85	275	2.60	50.0	90.0	8.0	119.4	1.34	id	id	[49]
2	Ellef Ringnes Island	Isachsen I-F	78.79	103.42	12	0.001	0.04	0.3	5.9	158.0	0.80	0.62	0.55	[50]
3	Ellesmere Island	Rock Basin	78.50	76.73	295	0.02	0.2	15.0	6.0	12.3	1.04	0.60	0.75	[51]
4	Melville Island	Pond MV-AT	75.32	111.42	120	0.10	0.4	0.4	8.1	39.0	0.88	0.92	0.64	[52]
5	Baffin Island	CF-11	70.47	68.67	96	0.11	0.6	11.0	6.9	42.7	1.23	0.53	0.86	[53]
6	Baffin Island	CF-10	70.43	69.12	435	0.09	2.7	9.6	6.4	23.9	0.77	0.62	id	[53]
7	Baffin Island	CF-3	70.53	68.37	27	0.20	0.6	7.2	5.9	68.9	0.74	0.60	0.69	[54]
8	West Greenland	Lake B	67.27	51.58	671	1.10	4.1	-	-	-	0.76	0.80	0.74	[55]
9	West Greenland	Nunutak	66.97	49.80	470	0.16	2.2	21.0	7.3	-	0.82	0.87	0.45	[55]
10	West Greenland	ss16	66.91	50.46	477	3.30	0.2	12.2	7.2	-	0.94	0.90	0.84	[55]
11	West Greenland	ss53	66.49	53.53	50	7.30	23.1	8.3	6.9	-	0.95	0.72	id	[55]
12	Northwest Territories	Slipper	64.62	110.83	460	1.90	134.0	17.0	6.6	31.2	1.01	0.88	id	[14]
13	Northwest Territories	TK-20	64.15	107.82	390	0.10	-	8.8	7.2	8.7	0.44	0.46	id	[14]
14	Nunavik (N Québec)	Airport	62.18	75.66	225	0.04	0.1	8.6	6.8	72.0	0.65	0.56	0.98	[56]
15	Nunavik (N Québec)	Tasikutaaq	62.16	75.72	50	0.31	8.6	13.6	6.5	23.0	0.64	0.67	0.88	[56]
16	Nunavik (N Québec)	Qaanganiittuq	62.12	75.59	342	0.06	0.4	13.9	-	-	0.67	0.57	0.48	[56]
17	Nunavik (N Québec)	Nipingngajulik	61.57	71.77	84	0.79	14.3	24.5	6.1	230.0	0.73	0.78	0.81	[56]
18	Nunavik (N Québec)	Allagiap Tasinga	61.54	72.01	156	0.21	3.8	9.0	6.0	21.0	0.75	0.82	0.97	[56]
19	Nunavik (N Québec)	Tasing	61.07	69.56	61	0.03	0.5	3.5	6.6	78.0	1.00	0.97	1.16	[56]
20	Nunavik (N Québec)	Lake X	60.86	70.13	128	0.31	20.4	6.5	7.2	80.0	0.59	0.63	0.54	[56]
										means:	0.84	0.71	0.75	
**Montane-boreal**														
21	BC Interior	Fraser	54.08	124.75	670	54.60	6707.1	30.5	7.5	-	0.60	0.79	0.59	[57]
22	BC Interior	North Barrière	51.33	119.83	634	4.50	517.7	52.6	6.6	-	0.51	0.42	0.53	[57]
23	BC Interior	Boomerang	53.68	124.58	1140	0.52	3.9	11.0	7.1	50.0	0.55	id	id	[58]
24	BC Interior	Secord	53.63	124.34	1220	0.40	2.2	8.0	7.0	55.0	0.57	id	id	[58]
25	BC Interior	Justine	53.85	125.09	820	2.48	43.7	10.0	7.1	59.0	0.50	id	id	[58]
26	BC Interior	Unnamed	53.85	125.09	1100	0.79	2.1	19.5	7.5	68.0	0.38	id	id	[58]
27	Vancouver Island, BC	Little Toquart	49.06	125.35	75	0.55	9.9	16.0	6.5	22.5	0.77	id	id	[59]
28	Vancouver Island, BC	Toquart	49.08	125.35	75	1.15	66.8	37.5	6.7	28.6	1.17	id	id	[59]
29	Vancouver Island, BC	Blue	48.73	124.90	90	0.47	5.3	11.0	7.0	24.3	0.37	id	id	[59]
30	Vancouver Island, BC	Angora	49.09	125.53	245	0.31	2.1	46.5	7.8	42.8	0.24	id	id	[59]
31	Northern Alberta	A86	55.68	111.83	678	75.00	197.0	2.7	6.6	25.7	0.86	0.64	id	[32]
32	Northern Alberta	L39	57.96	110.38	356	1.12	18.1	1.5	6.8	108.0	0.59	id	id	[32]
33	Northern Alberta	L107	59.72	110.01	366	3.73	12.2	7.8	7.3	59.7	0.63	id	id	[32]
34	Northern Alberta	L109	59.12	110.82	274	1.29	111.7	13.7	7.1	49.9	0.76	0.66	id	[32]
35	Northern Alberta	Legend	57.41	112.93	787	16.80	93.1	10.2	6.9	29.7	0.46	0.45	id	[32]
36	Northern Alberta	Namur	57.44	112.62	731	43.39	224.0	24.0	7.3	63.6	1.16	id	id	[32]
37	Northern Alberta	Otosan	57.71	112.39	750	3.44	23.4	7.6	6.8	24.7	0.55	0.68	id	[32]
										means:	0.63	0.60	0.56	
**Alpine**														
38	CDN Rockies (Jasper)	Curator	52.80	117.87	2232	0.05	0.8	25.0	8.4	220.0	1.15	0.72	0.46	this study
39	CDN Rockies (Banff)	McConnell	51.63	115.97	2300	0.08	2.7	31.0	-	-	0.68	0.62	0.39	this study
40	US Rockies (Beartooths)	Beartooth	44.95	109.60	2713	0.45	61.1	26.2	7.6	30.0	0.87	id	id	[60]
41	US Rockies (Beartooths)	Beauty	44.97	109.57	2874	0.36	15.0	35.1	6.9	7.0	0.82	0.33	0.28	[60]
42	US Rockies (Beartooths)	Emerald	45.00	109.53	3292	0.16	1.0	-	6.0	-	2.00	1.04	0.90	[60]
43	US Rockies (Beartooths)	Fossil	45.00	110.00	3018	0.67	4.1	45.7	6.7	6.0	0.52	0.38	0.38	[60]
44	US Rockies (Beartooths)	Heart	44.98	109.54	3162	0.16	0.9	45.7	7.3	6.4	0.56	0.27	0.33	[60]
45	US Rockies (Beartooths)	Island	44.95	109.54	2901	0.59	18.8	30.5	7.1	7.0	0.76	0.27	0.33	[60]
46	US Rockies (RMNP)	Sky Pond	40.28	105.67	3322	0.03	2.0	7.0	6.5	12.5	0.92	0.45	0.54	[4]
47	US Rockies (RMNP)	Snowdrift	40.34	105.73	3389	0.03	0.4	14.0	6.6	14.7	0.67	0.33	id	[4]
48	US Rockies (RMNP)	Husted	40.58	105.68	3350	0.04	1.5	11.0	6.9	13.4	0.88	1.08	id	[4]
49	US Rockies (RMNP)	Louise	40.55	105.62	3360	0.03	0.7	8.0	6.9	19.9	1.47	1.01	0.54	[4]
50	US Rockies (RMNP)	Nokoni	40.25	105.73	3292	0.08	0.8	38.0	6.4	10.4	0.86	0.49	id	[4]
51	US Rockies (Zirkels)	Pristine	40.69	106.68	3366	0.01	0.2	-	7.0	15.9	1.55	0.46	id	[16]
52	US Rockies (San Juans)	Crater	37.67	107.71	3546	0.03	0.4	13.4	7.0	39.0	1.48	1.17	1.00	this study
									means:	1.01	0.61	0.52		
									grand mean:	0.82	0.66	0.65		

- = not measured.

id = insufficient dating control.

BC: British Columbia.

RMNP: Rocky Mountain National Park, Colorado.

## Results

### Diatom Stratigraphies

**Figure 2:**
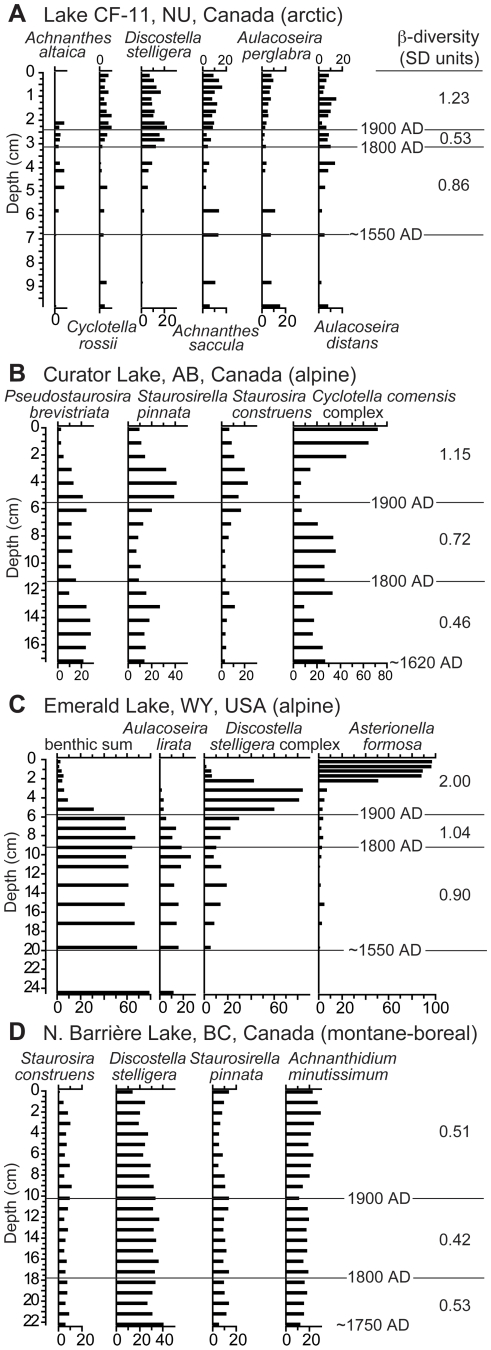
Examples of diatom stratigraphic records. Stratigraphies of diatom relative abundance are illustrated for one arctic lake (CF-11, A), two alpine lakes (Curator and Emerald, B and C), and one montane-boreal lake (North Barrière, D). Diatom taxa are ordered by descending DCCA axis 1 score from left to right. β-diversity values corresponding to each time interval are shown on the right. Only dominant taxa are shown, however all taxa >1% were included in β-diversity calculations.

Diatom β-diversity summarizes the amount of compositional turnover having occurred in a core's successive diatom assemblages over a specified interval of time. It is estimated in units of standard deviations (SD). We first illustrate examples of the raw data used in these computations by showing the relative frequencies of dominant diatoms in dated sediments from four lakes expressing variable amounts of assemblage change and representing a range of lake characteristics (Fig. 2). In the Arctic (CF-11, Baffin Island) and alpine lakes (Curator, Jasper National Park, Alberta, and Emerald, Beartooth Wilderness, Wyoming), the increased success of planktonic *Cyclotella* spp. and *Discostella* spp. in the 20^th^ century is evident (Fig. 2A–C). Both of these genera become competitive as the water column stratifies, and thus benefit from prolongation of the summer ice-free period [13]–[15]. During earlier intervals of lowered planktonic diatom abundance, small benthic taxa (e.g. *Pseudostaurosira*, *Staurosirella*, *Staurosira* and *Achnanthes* spp.) predominate in these lakes. These diatoms are successful when light attenuation is low, ice-free growing seasons are short, and production is concentrated in the littoral zone, often within ice-free moats [2], [9]. In these and other examples, it is the relative success of planktonic diatoms over benthic taxa in the 20^th^ century that drives β-diversity (Fig. 2).

An additional shift is observed in the sediments of Emerald Lake: after ∼1950, *Asterionella formosa* becomes completely dominant, replacing the *Discostella stelligera* complex as the principal planktonic diatom. The magnitude of changes in Emerald Lake's diatom flora produced the highest 20^th^ century β-diversity in our survey (2.00 SD). However, recent increases of *A. formosa* are in no way isolated, being relatively common in alpine lakes of the American Cordillera [4], where they have been linked causally to increase Nr availability from atmospheric deposition [16].

In contrast to lakes that preserve high (CF-11, Curator) to extreme (Emerald) degrees of diatom assemblage compositional turnover, a number of lakes possess relatively complacent diatom stratigraphies. Such lakes are commonly, but not exclusively, from the montane-boreal subset of sites (Table 1). For example, diatom assemblages in sediments from North Barrière Lake (British Columbia interior) have changed very little over recent centuries, resulting in low, near constant β-diversity (0.42–0.53 SD) over the length of the record (Fig. 2D).

### Diatom β-Diversity

**Figure 3:**
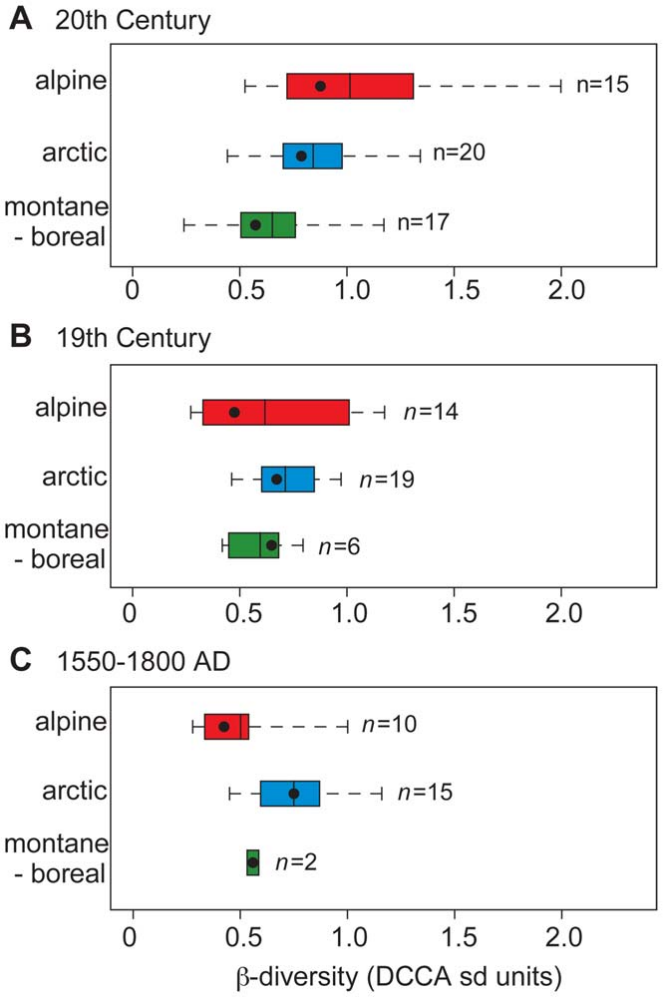
Boxplots of diatom β-diversity from each lake region. Black dots represent the median value, boxes are the 25^th^ percentile surrounding the mean value, and dashed ticks indicate range. (A) 20^th^ century results; statistically significant differences exist between alpine and montane-boreal (*p* = 0.003) and arctic and montane-boreal (*p* = 0.004) lakes. Results for the 19^th^ century (B) and 1550–1800 (C) reveal no statistically significant differences, in contrast to the 20^th^ century.

The estimated 20^th^ century β-diversities from both arctic (*p* = 0.004) and alpine (*p* = 0.003) diatom records are significantly higher than those from forested montane-boreal lakes (Fig. 3), as tested using Wilcoxon Rank Sum tests with Bonferonni-adjusted *p*-values [Materials and Methods]. The alpine sites range from 0.52–2.00 SD (mean: 1.01±0.43), the arctic sites range from 0.44–1.34 SD (mean: 0.84±0.22), and the montane-boreal sites range from 0.24–1.17 SD (mean: 0.63±0.27). However, during the 19^th^ century and the 1550–1800 intervals, β-diversities for arctic (*p* = 0.29 and *p* = 0.24, respectively) and alpine (*p* = 0.71 and *p* = 0.49, respectively) lakes do not differ significantly from montane-boreal counterparts (Fig. 3). Complete β-diversity results are reported in Table 1.

Additionally, 20^th^ century diatom β-diversities from alpine lakes are significantly greater than either interval of the prior 350 years (*p* = 0.01 for both time periods). These increase from a mean value of 0.52 SD during the LIA (1550–1800) to 0.61 SD during the 19^th^ century, rising to 1.01 SD in the 20^th^ century (Fig. 3A). A parallel increase in 20^th^ century diatom β-diversity is evident in the arctic lakes, with a lower level of significance (*p* = 0.05 for both time periods). Here, the succession of mean values is: 0.75 SD (1550–1800 AD); 0.71 SD (19^th^ century); and 0.84 SD (20^th^ century). In contrast, none of the temporal changes in diatom assemblage turnover from montane-boreal lakes represent statistically significant trends (*p* = 0.76 for the 19^th^ century and *p* = 0.89 for the LIA).

While we accept this result as a first approximation, we caution that the limited available dating control strongly limits inferences prior to the 20^th^ century from these sites, relative to the arctic and alpine records (Fig. 3, Table 1). Indeed, large changes in diatom assemblages from north-boreal lakes during the 19^th^ century have been documented elsewhere [14], [17].

Summarily, diatom β-diversity for all 52 lakes during the 20^th^ century is significantly greater than the 19^th^ century (*p* = 0.03), while there is only a small and non-significant difference in turnover between the 19^th^ century and 1550–1800 intervals (*p* = 0.86). Alpine and arctic lakes reveal greater diatom assemblage turnover in the 20^th^ century relative to forested montane-boreal sites, even though the previous 350 years reveal no significant differences between the three biomes (Fig. 3).

### Diatom β-Diversity – Environment Relationships

To investigate potential influences on diatom compositional turnover, we tested relationships between β-diversity and a number of physical and limnological characteristics of the lakes, including: lake and catchment area, maximum depth, pH, conductivity, [NO_3_^−^], modeled Nr deposition, gridded ambient summer and winter air temperatures, and modeled temperature trends 1948–2008 [Materials and Methods]. For each lake, we first standardized 20^th^ century β-diversity estimates to the 19^th^ century value, in order to account for between-lake differences in natural variability. We failed to uncover statistically significant relationships between standardized 20^th^ century diatom β-diversity and any one of the physical or environmental variables considered. For example, 20^th^ century diatom β-diversity is not correlated to lake-water pH (*r* = 0.01, *p* = 0.47). One reason there are no immediate correlations is the upward inflection of diatom β-diversity values at both extremes of the latitudinal gradient (i.e. arctic and alpine lakes, respectively), a pattern that is not mirrored by any single variable tested.

## Discussion

### Diatom β-Diversity, Latitude, and Altitude

**Figure 4:**
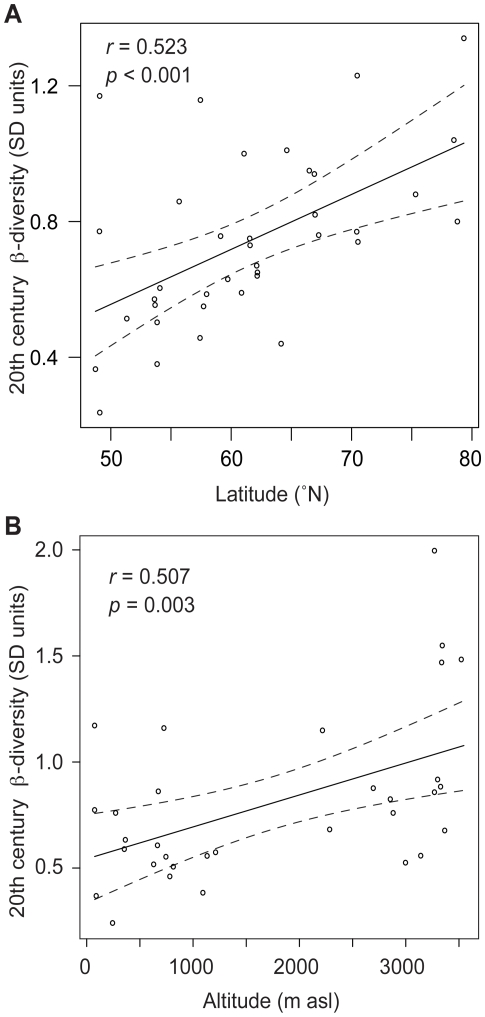
Scatterplots of 20^th^ century β-diversity against latitude and altitude. In (A), only the arctic and montane-boreal lakes are considered (*n* = 37), whereas (B) includes the alpine and montane-boreal sites (*n* = 32). Both relationships are highly significant (*p*<0.01; *p* = 0.03).

There are two geographic subsets within our data for which 20^th^ century diatom β-diversity is highest: high latitude-low altitude lakes and high altitude-low latitude lakes (Table 1). It has previously been demonstrated that diatom community turnover since 1850 increases with latitude in the Arctic [2]. Our present results confirm this finding, and places it in the longer context of the last 450 years. Furthermore, we show that 20^th^ century diatom turnover rates in high altitude lakes are of similar or greater magnitude to those recorded in the Arctic. When the data from arctic and montane-boreal lakes are collated (i.e. alpine lakes excluded), there is a significant linear relationship between 20^th^ century β-diversity and latitude (*r* = 0.52; *p*<0.001; *n* = 37, Fig. 4A). Similarly, when alpine and montane-boreal lakes are combined (i.e. sites >60°N excluded), a significant linear relationship (*r* = 0.51; *p*<0.003; *n* = 32) emerges between 20^th^ century β-diversity and altitude (Fig. 4B). In contrast, no relationships are evident between β-diversity and either altitude or latitude during the 19^th^ century or the LIA, indicating that these ecosystems do not have naturally high rates of diatom assemblage turnover. Thus, the observed relationships between diatom β-diversity and both latitude and altitude (Fig. 4) confer greater ecological responses among arctic and alpine lakes to 20^th^ century environmental changes than to natural perturbations, including the LIA cold spell.

**Figure 5:**
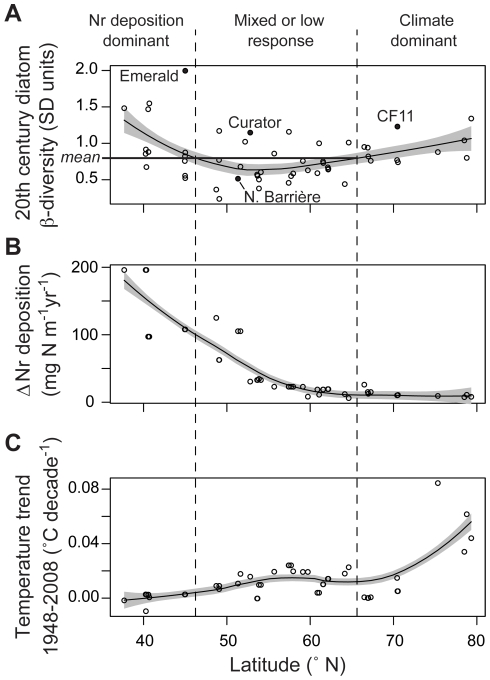
Latitudinal distribution of 20^th^ century diatom β-diversity in relation to modeled Nr deposition and recent climate change. In (A), the horizontal line represents the grand mean 20^th^ century β-diversity value (0.82 SD, *n* = 52), and vertical dashed lines represent the intersection of this value with the a LOESS smooth curve [48] fitted using a span of 0.75. Using the same approach, curves were fitted to the Nr deposition (B) and climate change (C) trends extracted from gridded data [Materials and Methods], revealing their respective latitudinal trends. The 95^th^ percentile confidence interval is shaded for each curve. Lakes illustrated in Fig. 2 are identified on panel (A).

These observed geographic patterns raise the hypothesis that 20^th^ century diatom community dynamics were driven alternately by changes in two external factors: climate warming and enhanced Nr deposition. Summarizing the 20^th^ century β-diversity trend over the complete latitudinal range of lakes in relation to the mean value (0.82 SD) enables a graphic delineation of the arctic and alpine regions characterized by the highest β-diversity values (Fig. 5A). When the climate change and Nr deposition gradients are added for comparison, Nr deposition rises for the alpine locations, while climate warming is greatest in the Arctic (Figs. 5B,C). Thus, the failure of 20^th^ century diatom β-diversity to correlate statistically with any individual physical or limnological variable, including surrogates for Nr deposition and climate warming, can be explained by the sensitivity of β-diversity to more than one primary forcing factor across the 52-lake population. Given the geographical breadth of lakes considered, this is perhaps not surprising. However, the reality of multiple stressors is their potential to act independently in some regions, and synergistically in others. For example, the diatom stratigraphy from Emerald Lake (Fig. 2C), which produced the highest 20^th^ century β-diversity of any site considered, reveals an early response consistent with warming (*Discostella* spp.) followed by later changes associated with Nr deposition (*Asterionella formosa*).

### Climate Change as a Driver of Diatom β-Diversity

**Figure 6:**
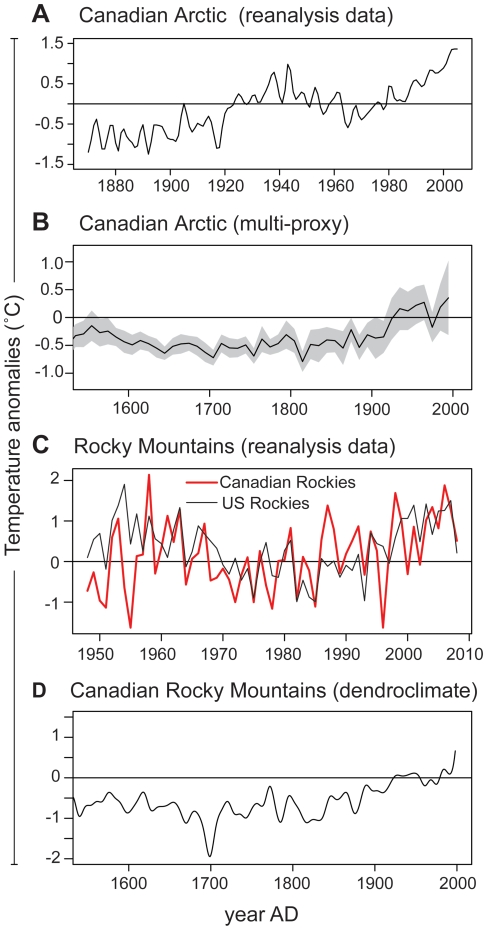
Temperature anomalies for arctic (A and B) and alpine (C and D) regions. (A) regional compilation of arctic (>60°N) instrumental temperature data for the period 1870–2005 (data from HadCRUTv2; http://www.cru.uea.ac.uk/). (B) Arctic multi-proxy paleoclimate reconstructions [18]. (C) NCEP/NCAR reanalysis data for alpine regions of the Canadian and American Rocky Mountains (data from http://www.cdc.noaa.gov/). (D) Alpine dendroclimatic reconstruction, showing temperature anomalies of the last 400 years [19]. The reanalysis data are expressed in relation to the 1961–1990 mean, whereas proxy reconstructions are calculated relative to 1901–1960. The shaded area in (B) indicates standard errors of prediction.

A compelling range of data reveals the legacy of pronounced climate warming during the last century, which is firmly imprinted in both arctic [18], [19] and North American alpine [20], [21] environments. Regional compilations of arctic temperature reanalysis data [22] and paleoclimatic proxies [18] suggest that mean temperatures since ∼1970 exceed any period within the prior 400 years (Fig. 6 A and B). The most recent arctic analysis [19] indicates that four of the five warmest decades of the past two millennia have occurred since 1950, and that recent summer temperatures are much as 1.4°C warmer relative to the long-term trend modulated by solar insolation. Furthermore, it has been established that the rate of arctic warming increases with latitude [23]. Although similar trends are evident in North American alpine regions, the rate and amplitude of warming is greater in the Arctic, primarily because positive feedbacks involving the cryosphere become accentuated with increasing latitude. Recent arctic warming corresponds closely with the regional increase of diatom β-diversity observed in high-latitude lakes over the 20^th^ century (Fig. 5), especially given that the character of diatom assemblage shifts in these lakes is consistent with those anticipated from warmer summers and longer growing seasons: increased diatom biomass, species richness and species size, and in many cases increased representation by planktonic forms [2].

However, it is important to note that climate trends in the Arctic are spatially heterogeneous [24], [25]. Accordingly, regions that have experienced the least recent warming, including northern Québec, Labrador, and west Greenland, also contain the arctic lakes with the lowest 20^th^ century diatom β-diversity values (e.g. Tasikutaaq Lake: 0.64 SD; Lake B: 0.76 SD, Table 1). This is concordant with the contention that climate warming is the primary driver of diatom β-diversity in the Arctic.

While climate change must be viewed as a potential driver of diatom shifts across each of the regions considered here, montane-boreal lakes tend to have considerably longer open-water seasons, so that thresholds for diatom taxonomic shifts are more gradually met. These ecosystems are potentially not as sensitive to small changes in climate or nutrient input [14], in part due to the edaphic stability of their catchments [26]. Relatively low mean 20^th^ century diatom β-diversity in these lakes (Fig. 3A) is entirely consistent with these attributes. Nonetheless, certain montane-boreal lakes produce very high 20^th^ century β-diversity, including Toquart Lake (Vancouver Island, 1.17 SD) and Namur Lake (northern Alberta, 1.16 SD). Such responses are, however, restricted to a minority of sites: 20^th^ century diatom β-diversity exceeds the grand mean of 0.82 SD in only 3 of 17 montane-boreal lakes considered (Table 1).

The impacts of recent climate warming on alpine lakes has been more closely investigated, revealing a range of hydrological and limnological changes that ultimately confer high degrees of sensitivity to climate change [21], [27]. Reanalysis data from the Rocky Mountains show similar late 20^th^ century temperature trends in both the Canadian and American sectors of the cordillera (Fig. 6C). The two warmest intervals of the 20^th^ century (1950–1970 and 1990-present) are the only decades that exceed mean 1901–1960 temperatures inferred from the 400 year dendroclimate record (Fig. 6D). Thus, recent warming in the Rocky Mountains is of lesser magnitude than that witnessed in the Arctic. And yet mean 20^th^ century diatom β-diversity from the alpine lakes is slightly higher than arctic counterparts (Fig. 3A), including the highest individual values and the only ones >1.4 SD (Table 1). In the few cases where analyses have been conducted in sufficient detail, the climatic legacy, while present, is less pronounced than that attributable to Nr deposition, with respect to both diatoms and stable isotopes [28]. The widespread recent proliferation of nitrophilous diatoms in American alpine lakes suggests that this assertion is regionally valid [4], [16]. The diatom succession from Emerald Lake aptly illustrates this type of complex response: *Discostella* spp. increase dramatically following the LIA, only to be swamped by *Asterionella formosa* after 1950 (Fig. 2C). It is on the basis of these observations that we consider in greater detail Nr deposition in relation to diatom community turnover in alpine lakes.

### Nr Deposition as a Driver of Diatom β-Diversity

Atmospheric emissions of Nr have increased 9-fold over the last century, now exceeding 140 Tg N yr^−1^ globally. Humans fix more N_2_ than the sum of natural processes [29]. In aquatic ecosystems, greater Nr availability from atmospheric subsidies becomes ecologically relevant by alleviating N limitation; lakes that are naturally N-limited or co-limited by N and P are thus most susceptible. It has been estimated that deposition rates in excess of 1.5 kg N ha^−1^ yr^−1^ are sufficient to induce rapid changes in the diatom communities of lakes [30]. However, a pronounced latitudinal gradient exists with respect to gridded estimates of Nr deposition increases between 1860 and 1993, mirroring patterns of human settlement and the intensity of agricultural activities [3]. With respect to the lakes under consideration here, the greatest increases in Nr deposition rates occur in the region immediately adjacent to low-latitude, high-altitude lakes of the Rocky Mountains (Fig. 5B). Here, 20^th^ century diatom β-diversity values are typically high, including the lakes in our survey that have changed the most Table 1). Given that climate warming is of lower amplitude than that witnessed in the Arctic, and considering the ecology of diatom taxa involved in these changes [4], [6], [16], [28], we surmise that Nr deposition has contributed directly to the inflection of 20^th^ century diatom β-diversity values observed in low-latitude alpine lakes (Fig. 5A). This is not to say that climate is unimportant in driving recent ecological changes in the alpine, but rather that the combined influences of climate warming and Nr deposition may conspire to produce higher 20^th^ century diatom β-diversities than those attributable to climate alone. The inclusion of additional alpine lake populations [1] is required to fully assess these nuances.

### Synthesis of Results by Principal Components Analysis (PCA)

**Figure 7:**
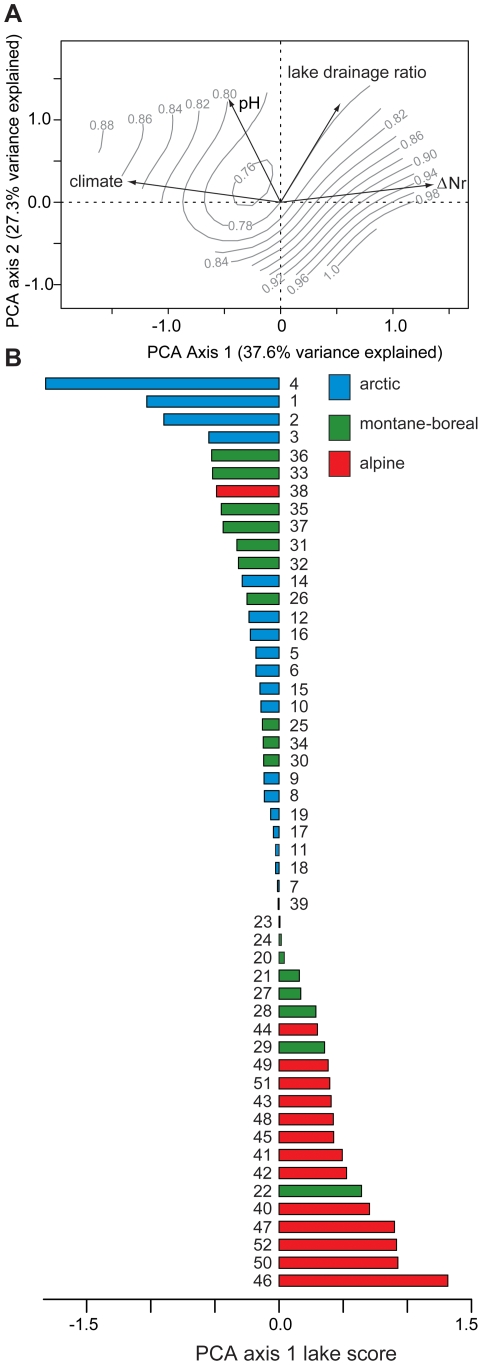
PCA of site-specific temperature and Nr deposition trends, lake pH, and lake∶catchment area ratio. (A) Biplot of the two first PCA axes, with 20^th^ century diatom β-diversity included in the PCA as a passive variable, contoured at 0.05 SD intervals using thinplate splines over the ordination space. (B) Lake scores ordered by PCA axis 1 score, with lake numbers corresponding to Table 1, and shading by region.

PCA allows an effective integration of both the climate and Nr deposition gradients for all 52 lakes, for which the additional variables of pH and lake:catchment area ratio are included to summarize the chemical and physical environments of each site. The independently-generated 20^th^ century β-diversity values are included in the analysis as passive variables, and subsequently contoured onto the resulting plot (Fig. 7A). The first PCA axis accounts for 38.1% of total variance explained, and conveniently distinguishes sites with high Nr deposition (positive scores) from those more influenced by climate warming (negative scores). This is the only significant principal component in the analysis, when compared to a broken-stick model based on randomly-generated matrices of identical proportions and total explanatory power [31]. The limnological variables (pH and lake:catchment area ratio) are more closely associated with the second axis, which is not statistically significant. This suggests that regional climate change and Nr deposition rates, which define the primary axis, are more important than site-specific factors in the analysis.

When 20^th^ century diatom β-diversity is passively superimposed on the PCA and contoured, values increase concentrically away from the origin (Fig. 7A). This implies greater diatom change at sites towards the extremities of axis 1, confirming that climate warming and enhanced Nr deposition alternately represent the dominant influence on diatom assemblage turnover within subsets of our lake population (Fig. 7A). The ordering of lake scores on the first PCA axis provides a visualization of site distribution along a gradient opposing climate change with Nr deposition (Fig. 7B). Although there are some exceptions, the arctic and montane-boreal lakes generally produce negative scores and hence are more strongly associated with temperature change, whereas alpine lakes have negative scores that confer a greater influence to Nr deposition. Montane-boreal lakes cluster more closely with the arctic population, suggesting that they have changed primarily in responses to climate warming. This is corroborated by recent increases in centric planktonic diatom populations in many of these lakes, and the absence of similar trends among taxa responsive to Nr deposition, such as *Asterionella formosa* [32]. The PCA thus provides a concise *a posteriori* verification of the pattern identified earlier: changes in diatom assemblages over the 20^th^ century relate to more than one environmental forcing, and these are expressed in a geographically coherent manner.

Finally, we note that lake scores on the primary PCA axis form a near-continuum of values (Fig. 7B), which mandates that some lakes are concommitantly influenced by climate change and Nr-deposition, even though their 20^th^ century β-diversity values may be modest. While we have attempted to separate these forcings, ultimately such efforts may prove futile because Nr delivery to lakes and climate warming are not mutually exclusive of each other. For example, climate warming influences precipitation, and in turn Nr deposition rates from wet deposition [33]. Furthermore, in both the Artic [34] and the alpine [26], melting of glaciers and perennial snowpacks has the potential to relegate anthropogenic Nr archived in snow and ice back to surface waters, irrespective of Nr deposition rates from precipitation. In our view, it is the potential for such synergistic processes that represents the most ominous threat for aquatic ecosystems in the 21^st^ century, given stratigraphic evidence that such combined changes are already underway in some regions (Fig. 2C).

### Conclusions

During the 20^th^ century, changes in diatom assemblages from both arctic and alpine lakes have accelerated relative to the previous 350 years. Elevated 20^th^ century β-diversity is associated primarily with climate warming and Nr deposition, with strong regional variations in the degree of influence attributable to either forcing factor. Given future scenarios for both climate change [35], [36] and Nr deposition [3], the diatom β-diversity trends are unlikely to be reversed, and in our view will only become exacerbated as the 21^st^ century progresses. Our study contributes further proof that distinctive biological fingerprints exist for the Anthropocene [37], while extending this notion to include the microbiota of remote lakes. As with organisms such as birds [38] and higher plants [39], future trajectories of lake diatom communities include states for which no prior analogs exist. Although the full range of ecological implications remain poorly understood, changes at the base of food webs necessarily entail consequences for higher trophic levels, while modifying the biogeochemical cycling of major elements including, but not limited to, carbon, nitrogen, phosphorus and silicon. Increases of primary production and organic matter sedimentation may also influence the recruitment of metals, both natural and anthropogenic, to sediments. The prognosis for truly unperturbed lake ecosystems, if indeed any still remain, is that they are highly susceptible to marked biological reorganizations. Lakes that have already entered new biological regimes will continue to change as humankind tightens its grip on both the global climate system and key biogeochemical cycles.

## Materials and Methods

### Site Selection, Core Chronology, and Diatom Analysis

Fifty-two diatom stratigraphies were compiled from lakes in western and northern North America and west Greenland (Fig. 1), spanning latitudes from 37.67°N to 79.33°N, and altitudes from 12 m asl to 3546 m asl (Table 1). The range of lake-water pH is 5.9–8.4, representing a large environmental gradient that is captured by diatom assemblages ranging, accordingly, from acidophilous to circumneutral to alkaliphilous. Sites can be categorized into arctic (>60°N; *n* = 20), alpine (above altitudinal tree-line; *n* = 15), and temperate montane-boreal lakes (<60°N and forested; *n* = 17) (Table 1). Despite being restricted to a single cordillera (the Rocky Mountains), the alpine lake population spans 15° of latitude, from southern Colorado to Alberta. The arctic lakes include sites from the continental Northwest Territories, much of the Canadian archipelago, northern Quebec, and west Greenland, together spanning 18° of latitude and 61° of longitude. To close the geographical gap (Fig. 1), and for meaningful comparisons with arctic and alpine sites, we also included a number of low elevation lakes in forested catchments of western Canada (Alberta, central British Columbia, and Vancouver Island). None of the lakes in our analysis is affected by direct point-source anthropogenic activities (e.g. shoreline development, effluent discharge, acidification). Most of the sites have been previously published upon in the context of local and regional studies (Table 1).

Continuous lake-sediment cores with an intact sediment-water interface were collected using a gravity-type corer [40]. Once recovered, cores were sampled continuously and immediately at either 0.25 cm or 0.5 cm resolution. Chronostratigraphy of the cores was established by ^210^Pb dating with alpha or gamma spectroscopy [41]. Sedimentation rates were extrapolated to an estimated age of 450 years only where constant stable values were reached in the unsupported ^210^Pb inventory of the core. Adequate chronology was the primary criterion for inclusion in our compilation. However, for many sites only the 20^th^ century is dated with acceptable accuracy and precision, so that the number of sites for which earlier intervals are considered is somewhat less than 52 (Table 1). The three sites with previously unpublished chronologies have unsupported ^210^Pb inventories that can be reliably interpreted using the constant rate-of-supply model (Fig. S1).

Preparation of sediment samples for diatom enumeration followed standard protocols [42]. Diatom enumeration was carried out on selected sediment intervals, at resolutions ranging from 0.25 cm to 3.0 cm. Two taxonomic complexes were used include small (<15µm) and ecologically comparable *Discostella* and *Cyclotella* spp., which can not be resolved by light microscopy: these are the ‘*Discostella stelligera* complex’ (*D. stelligera* and *D. pseudostelligera*) and the ‘*Cyclotella comensis* complex’ (*C. comensis*, *C. rossii*, and *C. tripartita*).

### Numerical Analyses

To estimate compositional turnover of sediment diatom assemblages, or β-diversity, relative abundances were analyzed using detrended canonical correspondence analysis (DCCA) constrained to time [12]. DCCA models assemblage composition as a unimodal response to one or more environmental variable. By constraining the DCCA to time, as inferred from ^210^Pb dating, we retain the biostratigraphic integrity of each sedimentary diatom sequence. The larger the β-diversity value obtained over the interval under consideration, the greater the assemblage turnover. This strategy allows for explicit comparisons between assemblage changes during the 20^th^ century and discrete earlier intervals. The selection of time slices for diatom β-diversity calculations is based on climate change over recent centuries. Paleoclimatic reconstructions consistently reveal the LIA (∼1550–1850 AD) as one of the coldest intervals of the Holocene, when glacial advances were widespread and low summer temperatures prevailed [43]. The onset of the LIA was gradual, and maximum glacier expansions occurred asynchronously between the late 17^th^ and 19^th^ centuries. The LIA terminated by the end of the 19^th^ century, and temperatures have increased since, although not monotonically. The latter part of the 20^th^ and the first decade of the 21^st^ century have probably witnessed the highest temperatures of at least the last millennium [44]. Accordingly, we adopted the following time slices for diatom β-diversity calculations: 1550–1900, the 19^th^ century, and the 20^th^ century. Owing to dating uncertainty and variable sediment recovery from site to site, the number of diatom records decreases with age (Table 1). Where dating control was adequate, we included only those lakes with a minimum of three sediment intervals within any one time period. The DCCA protocol included square-root transformation of relative frequencies to stabilize between-taxon variance, no down-weighting of rare taxa, inclusion of all taxa with >1% relative abundance, detrending by segments, and non-linear rescaling. DCCA was performed using CANOCO 4.51 [45].

We subsequently used multiple linear regression to test for correlations between selected physical and limnological variables and 20^th^ century diatom β-diversity. Variables include lake and catchment area, lake:catchment area ratio (a surrogate for water residence time), maximum depth, winter (December–February) and summer (June–August) ambient air temperatures, surface air temperature trends for each site from 1948–2008, measured lake-water pH, specific conductance, [NO_3_^−^], and modeled atmospheric reactive nitrogen (Nr) deposition between 1860 and 1993 [46]. Three gridded air temperature parameters were extracted for each lake's coordinates: (1) winter (December–February) and (2) summer (June–August) air temperatures from high resolution (10′) interpolations from station means for the period of 1961–1990 [25], and (3) surface air temperature trends 1948–2008 extracted from the NCEP/NCAR Reanalysis Project [22], summarized using the linear trend (slope) of the data period. Nr deposition estimates were extracted from gridded (5° longitude by 3.75° latitude) global data-sets using the nearest grid centroid to the corresponding lake coordinates [3], [46].

Non-parametric Wilcoxon rank sum tests were used to assess the statistical significance of β-diversity between lake categories within each time period and between time periods for each lake category. Levels of statistical significance were evaluated using Bonferroni-adjusted values of *p*. Finally, in order to summarize and evaluate collective environmental influences on β-diversity lake-by-lake, ordination by principal components analysis (PCA) was undertaken on the following standardized variables: 1948–2008 temperature trend, ΔNr deposition 1860–1993, pH, and lake:catchment area ratio (with 3 sites excluded due to insufficient catchment data). The latter two variables were included as conservative measures of lake chemical and physical characteristics, respectively. These analyses, as well as the extraction of gridded climate data, were conducted using the open-source software environment R [47].

## Supporting Information

Figure S1 210Pb chronology from three unpublished alpine lakes included in this study. Lakes are detailed in Table 1. All three age models met our criteria for inclusion in the study, yielding reliable dates through the unsupported 210Pb section of the core. Constant sedimentation rates near the limit of 210Pb dating are shown by the linear relationships of log 210Pb activity against cumulative dry mass and were used to extrapolate dates to approximately 1550.
(1.77 MB TIF)
