# Supplementary figures and images for: Quantifying Recent Ecological Changes in Remote Lakes of North America and Greenland Using Sediment Diatom Assemblages
Source: PLoS One. 2010 Apr 2;5(4):e10026. doi: 10.1371/journal.pone.0010026 (PMC2848865; doi:10.1371/journal.pone.0010026)

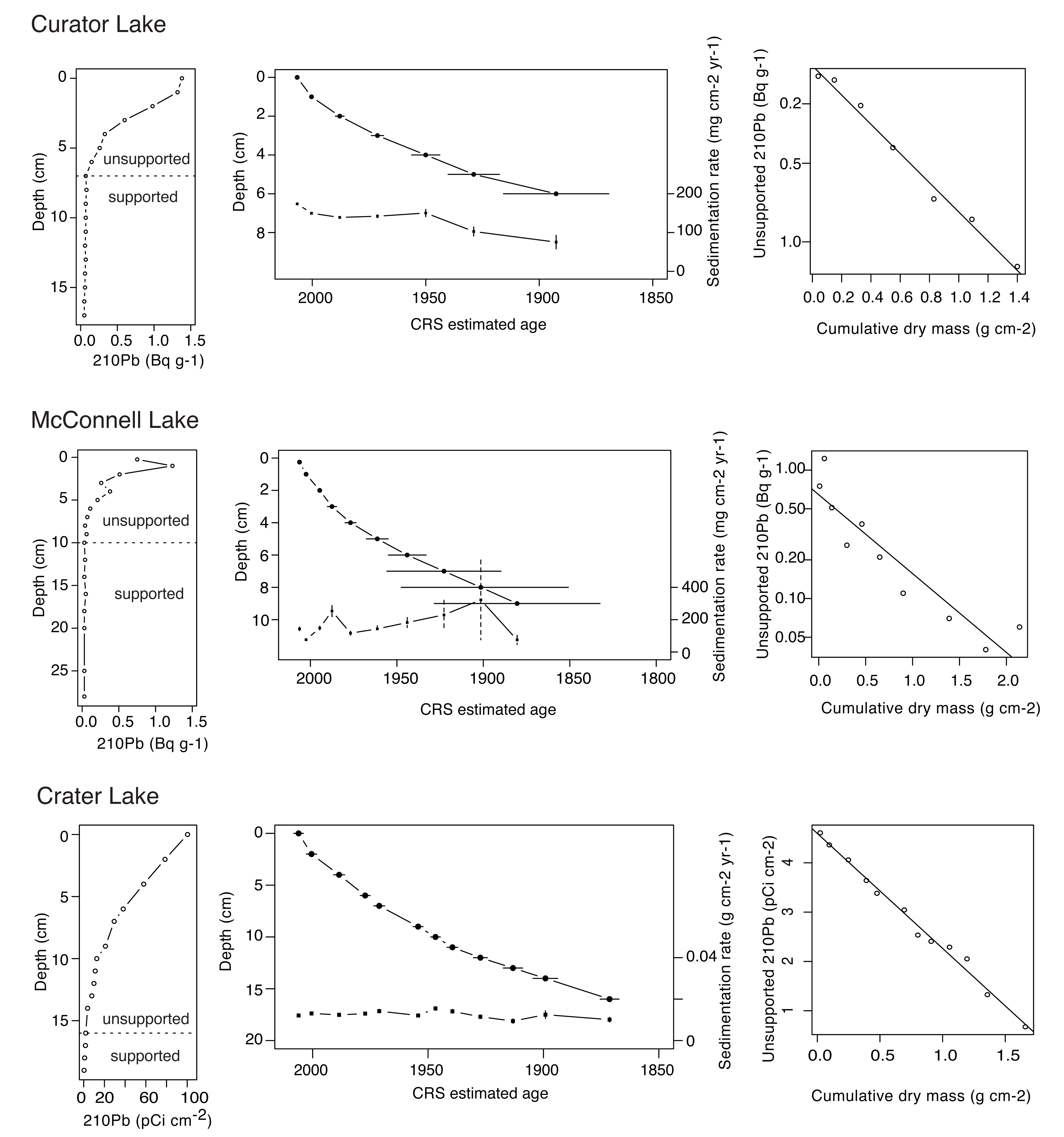

Supplement: Figure S1 — 210Pb chronology from three unpublished alpine lakes included in this study. Lakes are detailed in Table 1. All three age models met our criteria for inclusion in the study, yielding reliable dates through the unsupported 210Pb section of the core. Constant sedimentation rates near the limit of 210Pb dating are shown by the linear relationships of log 210Pb activity against cumulative dry mass and were used to extrapolate dates to approximately 1550. (1.77 MB TIF) [file pone.0010026.s001.tif]
